# Cell-Penetrating Peptides for Antiviral Drug Development

**DOI:** 10.3390/ph3030448

**Published:** 2010-03-02

**Authors:** Melaine Delcroix, Lee W. Riley

**Affiliations:** School of Public Health, University of California, Berkeley, CA 94720, USA; Email: mdelcroix@berkeley.edu (M.D.)

**Keywords:** cell-penetrating peptide, antivirals, antisense, drug delivery

## Abstract

Viral diseases affect hundreds of millions of people worldwide, and the few available drugs to treat these diseases often come with limitations. The key obstacle to the development of new antiviral agents is their delivery into infected cells *in vivo*. Cell-penetrating peptides (CPPs) are short peptides that can cross the cellular lipid bilayer with the remarkable capability to shuttle conjugated cargoes into cells. CPPs have been successfully utilized to enhance the cellular uptake and intracellular trafficking of antiviral molecules, and thereby increase the inhibitory activity of potential antiviral proteins and oligonucleotide analogues, both in cultured cells and in animal models. This review will address the notable findings of these studies, highlighting some promising results and discussing the challenges CPP technology has to overcome for further clinical applications.

## 1. Nature and Scope of the Challenges Presented by Viral Infections

Viral diseases affect hundreds of millions of people worldwide, resulting in a devastating toll on human health and socio-economic development. Along with the emergence of newly-recognized human pathogens (the SARS coronavirus, the recent influenza viruses H5N1 and H1N1), the ever-increasing incidence of chronic viral infections caused by HIV and hepatitis B and C viruses continues to increase the global burden of infectious diseases [1,2]. Vaccines have been developed for some of the most important viral pathogens. Although vaccines against HIV [3] and hepatitis C virus [4] are in clinical phases III and II respectively, there is still little prospect of effective vaccines against these agents. There are enormous challenges to the development of these vaccines, especially since millions of people are already chronically infected with these viruses, which would require therapeutic vaccines for control.

These challenges emphasize the importance of chemotherapy to treat these viral infections [5]. There are around 40 antiviral compounds in clinical use targeting various viral diseases (over half of these drugs are being used in the treatment of patients with HIV infection) while there is no treatment for most acute infections, such as the ones that cause severe illnesses, including hemorrhagic fever, encephalitis, and even cancer [6,7,8,9]. Most of the available drugs are of limited efficacy and come with severe side effects [6]. Importantly, antiviral chemotherapy is plagued by the rapid development of drug resistance strains, resulting from the high rate of replication of viruses combined with the low fidelity with which they replicate their genomes [10].

In view of the modest existing drug arsenal, the continuing threat posed by viral pathogens urgently calls for the development of novel antiviral agents. As obligate intracellular parasites, viruses present formidable challenges to drug development, the biggest being the *in vivo* delivery of the antiviral drug into the infected cells, with minimum toxicity to the host cells [6].

## 2. The Advent of Cell-Penetrating Peptides

Traditional antiviral therapy has relied on small molecules such as protease inhibitors or nucleotide analogues to inhibit viral enzymes. In the last decades, proteins and nucleic acid molecules have shown very promising antiviral properties. However, due to their physical properties, such as size or hydrophilicity, the cellular uptake of these molecules is strongly restricted. The inability of these molecules to cross the cell membrane to reach their intracellular target precludes further clinical application. The cellular delivery of these molecules has thus arisen as a cornerstone for therapeutic development.

Current techniques for cellular delivery of antivirals include targeted liposomes in cell culture and in mice [11,12,13,14], receptor-mediated endocytosis through antibody binding in cell culture and in mice [15], retroviral vectors in cell culture [16], as well as adenoviruses in vaccine delivery [17]. However these techniques present certain limitations and concerns: low efficiency [11,15], immunogenicity [14,15], stability and rapid clearance in bloodstream [18] for liposomal reagents, and immunogenicity and viral integration on host gene expression for viral vectors [15,19].

A novel strategy to efficiently overcome the impermeable cell barrier came from the surprising findings in the late 1980s that certain naturally occurring short peptide sequences have the ability to enter cells when added to culture media. The tat peptide, derived from HIV-1 transcriptional activator protein (tat) [20,21], and penetratin, derived from *Drosophila* antennapedia (Antp) transcription protein [22], were the first cell-penetrating peptides (CPPs) to be described. Tat and penetratin have paved the way to the discovery of other naturally occurring CPPs such as the herpesvirus tegument protein VP22 [23,24] or the cell wall protein-derived peptide inv3 from *Mycobacterium tuberculosis* [25,26]. Chimaeric CPPs such as transportan (a chimera of the neuropeptide galanin and the wasp venom toxin mastoparan) [27] and totally synthetic CPPs such as the model amphipathic peptide (MAP) [28] or arginine oligomers [29] have also been designed and are routinely used. The exact mechanism of cellular uptake is not clear and studies remain controversial. Current models include uptake through transient pore formation, caveolae, clathrin-dependent endocytosis, and macropinocytosis. Recent data suggest endocytosis as the prevailing model for uptake, although several mechanisms may coexist and differ depending on CPPs [30,31].

What makes these peptides very attractive as a delivery system is their ability to promote intracellular uptake of conjugated cargoes [32]. CPPs have successfully improved the cellular uptake of various cargoes including proteins [26,33], nucleic acids (oligonucleotides [34,35], peptide-nucleic acids [36,37], siRNAs [38,39]), nanoparticules [40] and liposomes [41] in a wide range of cells: mainly in mammalian cells, but also in bacteria [37,42], yeasts [42] and protozoan parasites [43,44]. CPPs have proven to be very efficient in delivering molecules into cells that are refractory to transfection such as primary lymphocytes [45]. In 1999, Schwarze *et al.* successfully delivered into all tissues in mice the 120-kDa tat-conjugated β-galactosidase protein, which retained its enzymatic activity [46]. Impressively, the conjugate had also crossed the blood-brain barrier and reached the brain tissues, which is usually restricted to small and highly lipophilic peptides. CPPs offer the opportunity to deliver therapeutic molecules that are 200 times larger than the current bioavailability size restriction [45]. CPP-mediated delivery of bioactive compounds into model organisms for cancer [47], cardiomyopathy [48], stroke [49], muscular dystrophy [50] and viral infections, support the strong therapeutic potential exerted by CPPs. Clinical trials are taking CPP-based drugs a step closer to therapeutic applications. An heptamer arginine-conjugated cyclosporine A (Psorban) has entered phase II of a clinical trial for topical treatment of psoriasis [29,51]. The oligoarginine peptide allows the penetration of cyclosporine A into cells throughout the otherwise impermeable epidermis and dermis.

This review will focus on studies that explored the use of CPPs to deliver antisense agents into virus-infected cells and animal models (Table 1).

**Table 1:** Antiviral agents successfully delivered into virus-infected cells *via* conjugation to CPPs. CPPs from conjugates which exhibited some antiviral activity are mentioned. R stands for arginine, X for 6-aminohexanoic acid, and B for β-alanine.

Antiviral cargo	Targeted virus	Conjugated CPP	Experimental systems	Limitations – CPP composition requirements
PMO	**RNA viruses**			
	*West Nile virus* [66,67]	(RXR)_4_XB	Cell culture	Dose-dependent toxicity in cell culture and mice
			Mouse	
	*Japanese encephalitis virus* [67]	(RXR)_4_XB	Cell culture	
	*St. Louis encephalitis virus* [67]	(RXR)_4_XB	Cell culture	
	*Dengue virus* [68,69,70]	(RXR)_4_XB	Cell culture	
		R_5_F_2_R_4_C	Mouse	
		R_9_F_2_C		
	*SARS coronavirus* [71]	R_9_F_2_C	Cell culture	
		R_5_F_2_R_4_C		
	*Mouse hepatitis virus* [59,63]	R_9_F_2_C (RXR)_4_XB	Cell culture Mouse	PPMO toxicity in mice when treatment given after MHV challenge

				PPMO with higher number of arginine residues exhibit greater antiviral activity in cell culture

				PPMO with insertions of 6-aminohexanoic acid offer greater protection in mouse
	*Equine arteritis virus* [72]	R_9_F_2_C	Cell culture	
	*Porcine reproductive and respiratory syndrome virus* [73]	R_5_F_2_R_4_C	Cell culture	
	*Poliovirus 1* [75]	R_9_F_2_C	Cell culture	*in vitro* toxicity when longer periods of treatment
		(RXR)_4_XB	Mouse	
	*Human rhinovirus 14*	R_9_F_2_C	Cell culture	
	[75]	(RXR)_4_XB		
	*Coxsackievirus B2*	R_9_F_2_C	Cell culture	
	[75]	(RXR)_4_XB		
	*Coxsackievirus B3*	(RXR)_4_XB	Cell culture	
	[76]		Mouse	
	*Foot-and-mouth disease virus* [74]	R_9_F_2_C	Cell culture	
	*Sindbis virus* [77]	R_9_F_2_C	Cell culture	
	*Venezuelan equine encephalitis virus* [77]	(RXR)_4_XB	Cell culture	
			Mouse	
	*Ebola virus* [64,78]	(RXR)_4_XB	Cell culture Mouse	PPMO with insertions of 6-aminohexanoic acid and higher number of arginine-6-aminohexanoic repeats offer greater protection in mouse
		R_9_F_2_C		
		(RX)_n=2-8_B		
		(RB)_8_B		
	*Respiratory syncytial virus* [79]	(RXR)_4_XB	Cell culture	Endosomal entrapment
			Mouse	
	*Measles virus* [80]	(RXR)_4_XB	Cell culture	
	*Influenza A virus* [81,82,83]	(RXR)_4_XB	Cell culture	Higher doses of PPMO induced abnormal infiltration of mouse lungs by immune system cells
		R_5_F_2_R_4_C	Mouse	
	**DNA viruses**			
	*Kaposi* *’s sarcoma-associated herpesvirus* [84,85]	R_5_F_2_R_4_C	Cell culture	
		(RXR)_4_XB		
	*Herpesvirus type 1*	(RXR)_4_XB	Cell culture	
	[86]		Mouse	
PNA	*HIV-1* [91,92,93,94,95,99,100,101]	**Disulfide-linked:**	Cell culture Some preliminary mouse studies for tat and penetratin conjugates	Endosomal entrapment requiring lysosomotropic agents Nature of CPP and of CPP-PNA linkage had an effect on conjugate activity
		tat		
		penetratin		
		transportan		
		transportan 21		
		transportan 22		
		R_6_-penetratin		
		**Stably-linked:**		
		tat		
		transportan		
		transportan 21		
	*Japanese encephalitis virus* [96]	tat	Cell culture	
siRNA	*Hepatitis C virus* [102]	tat	Cell culture	
	*HIV-1* [15]	nonamer arginine (9R)	Mouse	
		non-covalent binding		
Proteins	*HIV-1* [103,104,105]	tat	Cell culture	
	*Human papillomavirus type 18* [106]	9R	Cell culture	Nature of CPP directly impacted the level of antiviral activity
		PTD4		

## 3. Delivery of Antisense Agents

The past decade has witnessed the breakthrough of gene-silencing antisense agents including antisense oligonucleotides (AOS), AOS analogues, ribozymes, DNAzymes and the popular siRNAs. This novel approach is based on the discovery 30 years ago that nucleic acid molecules could be used to specifically target complementary viral nucleic acid sequences and inhibit viral replication [52,53]. With these new promising molecules comes the ability to simultaneously target multiple viral sequences, which would preclude the selection of drug resistance.

**Figure 1:**
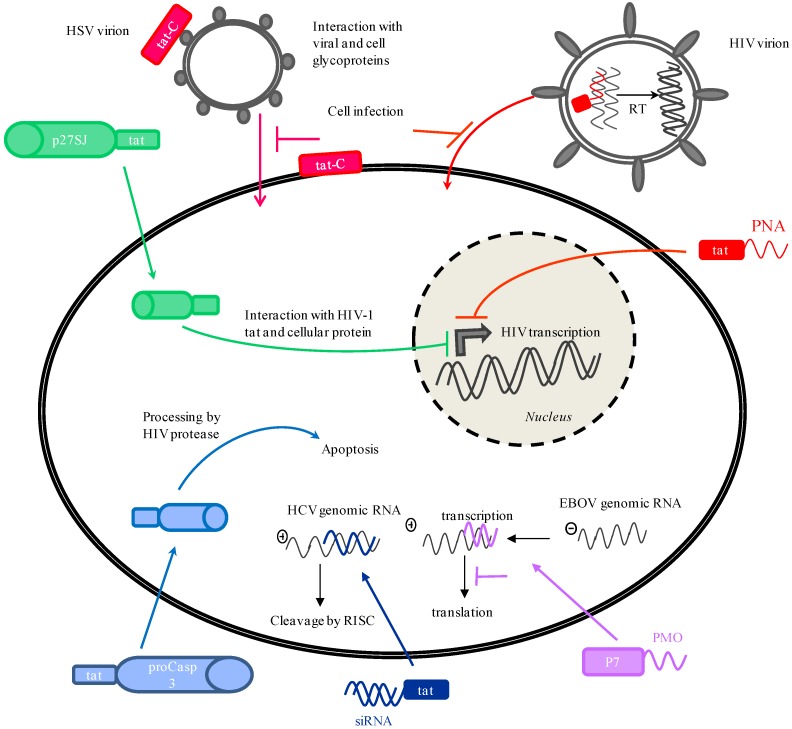
Mechanisms of antiviral activity exerted by CPP conjugates. These antiviral agents can target retroviruses, as shown for HIV, non-retroviral RNA viruses, as shown for the negative-sense RNA EBOV and HCV, and DNA viruses, as shown for HSV. Interference with viral RNA processing and translation further inhibits viral replication. CPP conjugates can also be designed to target virus-infected cells. CPP can also inactivate the virion and render cells resistant to infection, inhibiting cell infection. proCasp3 stands for pro-caspase 3; RT stands for reverse transcription; Tn stands for transportan.

### 3.1. Delivery of Phosphorodiamidate Morpholino Oligomers (PMOs)

So far, conjugation of CPPs to phosphorodiamidate morpholino oligomers (PMOs) has clearly been the most popular and most promising use of CPPs in antiviral development. PMOs are antisense DNA oligonucleotide analogues [54]. Their backbone is composed of morpholine rings joined by uncharged phosphodiamidate linkages, in place of the sugar and anionic phosphodiester linkage of DNA. They are typically synthesized as 20–25-base long oligomers. They offer great features for clinical applications: they are water-soluble, nuclease-resistant, and their uncharged backbone interacts weakly with serum and cellular proteins thereby reducing toxicity [55]. PMOs are mainly designed to interact with 5’ and 3’ non-coding regions of the target mRNA, AUG-codon translation start-site region or splice junctions. They act through steric blockage of the complementary RNA sequences, precluding proper mRNA processing or translation and thus reducing target protein levels and viral replication (Figure 1).

As PMOs do not efficiently enter cells on their own, investigators have been routinely arming them with CPPs to promote their uptake into virus-infected cells and enhance their antisense efficacy [56,57]. These peptide-conjugated PMOs are referred to as PPMOs in the literature. As highlighted by Moulton and Jiang [58], PMOs are particularly well-suited cargoes for CPPs as their uncharged components do not interact with the positively-charged carrier peptide thereby not interfering with its membrane binding properties. The first report of PPMO antiviral activity in 2004 [59] has fueled the increasing interest in PMO-technology to inhibit viral infections as witnessed by more than 20 *in vitro* and/or *in vivo* studies published on antiviral PPMOs within the last five years (Table 1). Studies on RNA viruses have been extensively reviewed by Stein [60] with the latest *in vivo* work discussed by Moulton and Jiang [58]. We will focus here on the overall findings in regards to the cell penetrating moiety of the conjugates, and we will then review the studies on targeting DNA viruses.

#### 3.1.1. Nature of Peptides Conjugated to PMOs

Peptide-mediated PMO delivery has been the focus of intense efforts to identify active, stable, non toxic and endosomolytic CPPs, of which (RXR)_4_XB (also called P7; R = L-arginine, X = 6-aminohexanoic acid; B = β-alanine) has proven very successful [61,62]. In antiviral studies, PMOs have been covalently linked to various arginine-rich peptides, the most frequent being R_9_F_2_C (R = L-arginine; F = L-phenylalanine; C = L-cysteine), R_5_F_2_R_4_C (also called P4), and (RXR)_4_XB. Conjugation is mainly at the 5’-terminal end of the PMO. In studies comparing the activity of various CPPs against murine hepatitis virus (MHV) [63] and Ebola virus (EBOV) [64], arginine-rich peptides with insertions of 6-aminohexanoic acid stood out for their *in vivo* effectiveness. The higher the number of arginine-6-aminohexanoic repeats, the higher were PMO antiviral effect on MHV in cell culture [63] and the protection against EBOV infection in mice [64]. These findings agree with the results from Amantana *et al.* [57], Abes *et al.* [61] and Youngblood *et al.* [65], who also found that these residues increased serum stability and enhanced endosomal escape of the conjugates. Burrer *et al*. documented that the nature of the linker (XB or cysteine) did not affect PPMO *in vitro* effectiveness against MHV or cytotoxicity, although the insertion of β-alanine residues was reported to improve both serum and intracellular stability of PPMO conjugates [65].

#### 3.1.2. PPMOs against RNA Viruses

PPMO antiviral activity has mainly been exploited against a wide range of non-retroviral RNA viruses. In cell culture, PPMOs were tested against West Nile virus (WNV) [66,67], Japanese encephalitis virus [67], St. Louis encephalitis virus [67], dengue virus (DENV) [68,69,70], severe acute respiratory syndrome coronavirus (SARS-CoV) [71], MHV [59,63], equine arteritis virus [72], porcine reproductive and respiratory syndrome virus [73], foot-and-mouth disease virus [74], poliovirus 1 (PV1) [75], human rhinovirus 14 [75], coxsackievirus B2 [75], coxsackievirus B3 (CVB3) [76], Sindbis virus [77], Venezuelan equine encephalitis virus (VEEV) [77], EBOV [64,78], respiratory syncytial virus (RSV) [79], measles virus [80], and influenza A virus (FLUAV) [81,82].

Cell culture assays consistently showed that PPMO readily entered infected cells and could specifically and strongly inhibit viral replication. Fluorescein-labeled PPMOs accumulated in the nucleus but were present throughout the cells [66,79]. Lai *et al*. [79] reported punctuate foci of signal in the cell cytoplasm which might be indicative of endosomal entrapment of PPMO.

Many investigators identified potent PPMO activity in cell culture assays and went on to test their antiviral properties in murine experimental models. Most of the investigators explored both the prophylactic and therapeutic potential of PPMO and the best protection was usually achieved with pre-infection and post-infection PPMO treatments. PPMOs were shown to suppress viral replication, attenuate symptoms of disease in and/or increase survivorship of mice infected with WNV [67], DENV [70], MHV [63], PV1 [75], CVB3 [76], VEEV [77], EBOV [64,78], RSV [79], and FLUAV [82,83]. Some PPMOs demonstrated very profound and impressive antiviral effects. In particular, two 5-μg pre-infection doses of (RX_8_)B-conjugated PMOs completely protected mice against lethal challenge with EBOV [64,78]. Mice treated with 200-μg doses of a (RXR)_4_XB-conjugated PPMO given pre- and post-infection survived VEEV lethal infection [77]. The authors found that over the 28-day period of monitoring, VEEV was undetectable in PPMO-treated mice brain, blood and peripheral tissues.

#### 3.1.3. PPMOs against DNA Viruses

While PPMOs are being routinely tested against RNA viruses, there have only been a handful of studies documenting the use of PPMO technology against DNA viruses. The first report came from Zhang *et al.* who were interested in blocking Kaposi’s sarcoma-associated herpesvirus (KSHV) lytic replication [84]. They explored the effects of the inhibition of the expression of KSHV latency-associated nuclear antigen (LANA) and replication and transcription activator (RTA), two factors involved in latency maintenance and in the switch to lytic phase, respectively. To do so, the investigators designed PMOs directed against LANA and RTA and covalently conjugated them at their 5’ terminal to R_5_F_2_R_4_C peptide. While fluorescein (Fl)-labeled PMOs entered cells poorly, Fl-PPMOs were efficiently taken up by BCBL-1 lymphocytes which, as emphasized by the authors, are difficult to efficiently transfect with common techniques such as lipofectamine. PPMOs could effectively inhibit RTA and LANA protein expression in cell culture. Treatment of KSHV-infected cells with RTA PPMO suppressed KSHV lytic replication. RTA knock down resulted in reduced levels of proteins expressed downstream of RTA. One of these downstream proteins is viral interleukin-6 (vIL-6) and is thought to be essential for the development of KSVH-associated diseases. In a subsequent study, Zhang *et al.* blocked the expression of vIL-6 with PPMOs. vIL-6 PMOs were covalently conjugated at their 5’ to (RXR)_4_XB or R_5_F_2_R_4_C. PPMO treatment inhibited vIL-6 expression, which in turn reduced human IL-6 level and KSHV yield, and reduced growth of treated cells [85]. 

Moerdyk-Schauwecker *et al.* tackled the reactivation of another DNA virus, the herpes simplex virus type 1 (HSV-1) [86]. They investigated the antiviral activities of five (RXR)_4_XB-conjugated PMOs directed against three HSV-1 immediate-early genes, ICP0, ICP4 and ICP27 crucial for HSV-1 replication. The most potent PPMOs designed against ICP0 or ICP27 strongly inhibited HSV-1 replication in cell culture by reducing viral protein expression (Figure 1). In particular, ICP0 PPMO was able to suppress the replication of several HSV-1 strains, including an acyclovir-resistant strain. The authors also tested ICP0 PPMO in a mouse model of ocular herpes infection. Topical application of 10 μg ICP0 PPMO to the eyes of HSV-1 infected mice inhibited virus replication in mouse eyes and reduced the incidence of eye disease by 37.5–50%, thereby preventing death associated with HSV-1 eye infection. To evaluate *in vivo* PPMO toxicity, the investigators applied 100 μg ICP0 PPMO (ten times the antiviral dose) daily to the eyes of uninfected mice. No gross or microscopic eye damage, no effect on body weight and temperature and no change of behavior were observed following the seven-day treatment. These results strengthen the potential of PPMOs as a treatment for recurring corneal disease from HSV-1 reactivation. 

#### 3.1.4. Enhancement of PMO Antisense Activity

Besides improving PMO cellular uptake, the CPP moiety was also shown to intensify PMO antisense activity against EBOV by 10- to 100-fold in cell-free translation assays [64]. These results are consistent with the findings of Nelson *et al.* who showed that PPMOs exhibited antisense activity 3- to 25-fold higher than corresponding PMOs while bearing lower off-target effects [87]. The authors propose that the arginine-rich peptides enhance RNA-PMO binding affinity thereby increasing specific antisense activity. 

#### 3.1.5. PPMO Toxicity

PPMOs appear to be generally well tolerated by cultured cells and animal recipients. However, some studies reported toxicity in cell culture with increase of PPMO dose [66] or longer period of exposure [66,75], and *in vivo* toxicity as the treatment dose increased, usually at doses higher than antiviral ones [67]. The peptide moiety of PPMOs seems to contribute to this toxicity. When Deas *et al.* tested both PMO and PPMO against WNV in mice, they found that mice could tolerate higher doses of PMO compared to PPMO (3 mg against 300 μg, respectively) [67]. Mice treated with high doses of PPMO would suffer from weight loss and develop abnormal behavior and smaller livers. Besides dose-dependent toxicity, the schedule of treatment regimen may also affect PPMO toxicity: while Burrer *et al.* did not document any treatment-associated toxicity when PPMOs were administered to healthy mice, they observed significant toxicity when PPMO treatment was given after MHV challenge [63]. Lupfer *et al.* reported abnormal infiltration of mouse lungs by immune system cells when PPMO targeting FLUAV was administered at higher doses [83]. The authors hypothesized that the arginine-rich CPP mimics the eosinophil major basic protein, also rich in arginine residues, in triggering the migration of inflammatory cells.

#### 3.1.6. PPMO *vs*. PMO *in vivo* Antiviral Efficacy

There have been a few reports of PMO-mediated antiviral activity in animal models. How PMOs could successfully suppress viral replication *in vivo* without the help of CPPS is not understood. Warfield *et al.* reported the nearly complete protection of rhesus macaques against EBOV lethal infection using PMOs [88]. PMOs were also reported to increase the survival of calicivirus-infected cats [89]. Studies have been done that directly compared PMO and PPMO antiviral activity *in vivo*. One study found that both PPMO- and PMO-treated mice survived EBOV lethal challenge (100% and 85% respectively, the difference being not statistically significant) [78]. However, other comparative reports clearly document that PPMO are far more effective than PMOs in mice. PPMOs offered 100% protection against EBOV infection at 5-μg and 50-μg doses while PMOs gave only 15% protection at 50-μg doses [64]. PPMOs increased survival of DENV-infected mice up to eight days whereas PMOs did not provide any survival benefit [70]. PMOs could not protect mice against WNV infection while PPMOs, even at doses 10-fold lower, delayed clinical signs and increased WNV-infected mice survival by 60% [67]. The differential activities observed in EBOV studies may be explained by the fact that Swenson *et al*. used the (RXR)_4_XB peptide while Enterlein *et al*. linked their PMO to the R_9_F_2_C peptide.

### 3.2. Delivery of Peptide-Nucleic Acids (PNAs)

Peptide-nucleic acids or PNAs are oligonucleotide analogues in which the sugar-phosphate backbone has been replaced with peptide bonds [90]. They are resistant to both nucleases and proteases. They are used as antisense molecules to specifically bind to their complementary DNA or RNA sequences, creating a structural hindrance and inhibiting viral transcription, translation and replication processes.

#### 3.2.1. Antiviral Activity of CPP-PNA Conjugates in Cultured Cells

Reports of anti-HIV-1 PNA delivery *via* CPPs come from Pandey’s [91,92,93,94] and Gait’s [95] groups which have been working on antivirals targeting highly-conserved regulatory regions of the HIV-1 genome critical for its replication and gene expression, such as the primer-binding site, the A-loop and the transactivation response element (TAR). In their studies, the authors designed PNAs directed against these regions to inhibit HIV-1 gene expression and replication (Figure 1).

The poor cellular uptake of these uncharged compounds led Pandey and co-workers to covalently conjugate PNA at its 5' end to various cell-penetrating peptides, including transportan, tat and penetratin, *via* disulfide bridges [91,92,93,94]. Upon coupling to these carrier peptides, the PNA cargoes were rapidly taken up by different human cell types in culture. When added to culture medium, these anti-HIV-1 PNA–CPP conjugates effectively inhibited HIV-1 replication, tat-dependent trans-activation and viral production by infected cells; no significant decrease was observed with the unconjugated PNA, emphasizing the great promise held by these conjugates as antiviral agents [91]. 

Turner *et al.* also chose to enhance the cellular delivery and biological activity of PNA by covalently attaching them to CPPs [95]. They raised the more technical but nonetheless very important question of how the type of CPP and the way it is linked to the PNA affects the ability of the PNA to reach its cellular target. They conjugated the PNA to the CPP either via a stable polyether linker or through a cleavable disulfide bond and tested a range of CPPs. They could inhibit tat-dependent trans-activation in HeLa cells with disulfide-linked conjugates of PNA to transportan or to the chimeric peptide R_6_-penetratin (R = L-arginine). However, unlike what was reported by Tripathi *et al.* [93], they found that tat and penetratin conjugates failed to inhibit transcription. None of the conjugates carrying the stable polyether linker could elicit trans-activation inhibition. Interestingly, supplementation of cell medium with the lysosomotropic reagent chloroquine enhanced inhibitory activity of disulfide-linked conjugates and stably-linked conjugates gained some inhibitory activity. Supported by confocal microscopy data on cell localization of conjugates, the authors argued that endosomal release is a limiting factor in conjugates efficiency: following cell uptake, CPP-PNA conjugates would be entrapped in endosomal or other membrane-bound cytosolic compartments and some of them would then be slowly released. Endosomal entrapment was also reported with PPMO conjugates [79]. However, this observation differs from Chaubey *et al.* conclusion of the conjugates not being taken up by the endocytic pathway as uptake was not affected by low temperature or phenylarsine oxide [92]. Turner *et al.* attempted to improve not only the cell delivery but also the endosomal release properties of CPPs to increase PNA antiviral activity [95].

As no effective therapy is currently available for treatment of JEV infection, Yoo *et al.* investigated the antiviral activity exerted by various PNAs directed against JEV RNA *cis*-acting elements [96]. All PNAs were conjugated to tat *via* an O-linker to promote efficient cellular uptake. Tat-PNAs successfully inhibited JEV proliferation in cell culture. JEV replication is thought to take place in specialized structures derived from host membranes harboring the viral RNA polymerase and host proteins involved in viral RNA replication [97]. This would mean that not only tat-PNA conjugates crossed the plasma membrane but they were also able to penetrate into the convoluted membrane system to interfere with viral replication.

#### 3.2.2. Preclinical Studies of CPP-PNA Conjugates in Mice

To date, only few *in vivo* studies have been reported with CPP-PNA conjugates [98]. In the field of antivirals, Pandey and colleagues have started preliminary toxicity, immunological and pharmacokinetic studies in mice for the anti-HIV-1 PNA_TAR_-penetratin conjugate [99,100,101]. Chaubey *et al.* reported the non-toxicity of the complex when administered at repeat doses ranging up to 100 mg/kg [99]. Mice which were given 100 mg/kg of the conjugate, a dose the authors highlight is far in excess of the expected therapeutic dose, suffered from diarrhea and reduced physical activity and from spleen, liver and kidney serotosis. However they recovered during the 60-day follow-up period with no irreversible organ damage reported.

Upadhyay *et al.* demonstrated that PNA_TAR_-penetratin conjugate is moderately immunogenic mainly due to its penetratin moiety [100]. Cytokine secretion profiles of the lymph node cells showed elevated levels of proinflammatory cytokines, such as IL-2 and IL-12, which are known to promote proliferation of T lymphocytes and slow down the death of CD4+ T cells. The authors emphasize that these immunogenic properties of the conjugate could prove to be beneficial to the host.

Ganguly *et al.* studied the tissue distribution and clearance of ^125^I-labeled PNA_TAR_ and its penetratin and tat conjugates [101]. They reported the distribution of the conjugates throughout the mouse major internal organs when administered by oral route as well as their slow release and clearance from different organs. Surprisingly, unconjugated naked PNA_TAR_ displayed a similar tissue distribution and clearance profile although the extent of its uptake was lower than its CPP conjugate. *In vivo* antiviral activity of PNA_TAR_ and its penetratin and tat conjugates needs to be assessed.

### 3.3. Delivery of Small Interfering RNAs (siRNAs)

The use of CPPs to deliver siRNAs into cells has rather been limited to date, even more so for antiviral siRNAs. Järver *et al.* suggested that siRNAs could be less amenable to CPP delivery due to charge interactions between the peptide and the siRNA and inefficient endosomal escape of the conjugates [30]. To our knowledge, the only report of CPP-mediated delivery of antiviral siRNA is from Meng *et al.* who constructed a cell-permeable siRNA targeting hepatitis C virus 5’ untranslated region [102]. The siRNA was crosslinked at its 3’ end with the tat peptide. The authors reported the cellular uptake and antiviral effect of the CPP-siRNA as being as efficient as transfection with lipofectamine in Huh-7 cell culture. However, some cell types being refractory to lipofectamine transfection which can moreover be toxic *in vivo*, CPP-mediated delivery of siRNA is definitely worth investigating in other cell types and in animal models of viral infections.

Although Kumar *et al.* did not use CPP as a cell delivery system *per se*, it is noteworthy that they exploited its binding capability to polyanionic nucleic acids such as siRNAs [15]. The authors developed a novel method for systemic delivery of antiviral siRNAs into T cells in mice: they used an antibody to the CD7 receptor as their delivery system by receptor-mediated endocytosis. They bound the siRNA to the antibody through the positively charged nona-D-arginine (9R) peptide conjugated to its C-terminal *via* a disulfide bond. They successfully suppressed HIV-1 infection in a humanized mouse model using a cocktail of these siRNA complexes, inhibiting HIV-1 entry into target cells and its replication. 

## 4. Delivery of Antiviral Proteins

The following studies have harnessed the carrier capability of the tat and 9R peptides to shuttle potential antiviral proteins into virus-infected cells. To our knowledge, the first report of the successful use of CPP to deliver potential antiviral agents into cells is by Vocero-Akbani *et al.* in 1999 [103]. In their elegant study, the authors took a novel approach to specifically kill HIV-infected cells thereby preventing the production of infectious virions. They engineered a zymogen form of the apoptosis-promoting enzyme caspase 3 that, upon entry into HIV-infected cells, would be processed into its active form by the HIV protease (Figure 1). To deliver this modified caspase-3 into Jurkat T cells, they armed the recombinant protein with the tat peptide at its N-terminal. The authors showed that they could transduce about 100% of cells in less than 20 min. Confocal analysis localized FITC-labeled TAT-caspase-3 to both cytoplasmic and nuclear compartments. While TAT-caspase-3 remained inactive in uninfected cells, its uptake resulted in massive apoptosis in HIV-infected Jurkat T cells. Although a very promising study, no further or follow-up investigations have been reported.

Due to its ability to interact with cellular and viral proteins, p27SJ, a protein from the St John’s wort, has been put forward as a potential anti-HIV-1 molecule by Darbinian *et al.* [104]. Recently, the investigators have used the tat peptide as part of a bi-directional protein transduction system where the antiviral protein of interest, p27SJ, was expressed with tat at its C-terminal. Secreted into the cell medium, the fusion protein efficiently entered HIV-infected cells through a tat-mediated uptake and strongly inhibited HIV-1 transcription and replication (Figure 1).

The tat peptide was also conjugated to peptide ligands impairing interactions between the HIV-1 regulatory protein Rev and host cellular factors [105]. Following their tat-mediated cellular uptake into primary lymphocytes and macrophages, these inhibitory heptapeptides, expressed in fusion with stabilizing proteins, successfully suppressed HIV-1 replication, although at a concentration higher than those of currently used antiviral drugs. Along with a better design of the peptide ligand, conjugation to other CPPs may improve cellular uptake and therefore antiviral activity.

In addition to targeting HIV-1, CPP-mediated protein delivery was further exploited to inhibit human papillomavirus type 18 (HPV-18) in cell culture [106]. Mino *et al.* delivered artificial zinc-finger proteins (AZPs) into cultured cells by expressing AZPs in fusion with the 9R peptide. These AZP-9R conjugates strongly inhibited HPV-18 replication to 3% at 250 nM while gene-delivered AZP only achieved 12% inhibition. PTD4 conjugates were also tested but proved to be less effective than AZPs fused to 9R.

## 5. Antiviral Properties Exerted by CPPs Themselves

While studying the delivery of anti-HSV-1 antivirals using a peptide derived from FGF4 signal peptide, Bultmann *et al.* found that the peptide itself exerted antiviral activity [107]. This discovery led them to investigate the intrinsic antiviral properties of a range of peptides against HSV-1 infection in cultured cells. Tested peptides included EB (stands for entry blocker; it is a modified FGF4 signal peptide), tat, KLA (a synthetic amphipatic peptide), and HOM (derived from *Drosophila* antennapedia protein), and their modified peptides [107,108,109]. In particular, a tat peptide containing a cysteine residue at its C-terminal (tat-C) was shown to be able to inhibit infection in three ways: irreversibly inactivating virions exposed to tat-C prior to cell infection, blocking entry of cell-adsorbed viruses, and inducing a state of resistance to infection in cells pretreated with tat-C (Figure 1) [109]. Mechanisms of antiviral activities have not been determined yet but tat-C seems to act by inhibiting viral entry at the fusion step rather than at the virus attachment step. Virion inactivation may result from tat-C binding to sialic acids on the viral envelop glycoproteins.

Along with the antiviral property of their anti-HIV-1 PNA-CPP complexes, Chaubey *et al.* and Tripathi *et al.* investigated the virucidal effect of their conjugates and their potential use as microbicide [92,94]. They showed that pre-incubation of HIV-1 virions with these molecules rendered them noninfectious and blocked further cell infection. Two hypotheses may explain these results (Figure 1). The conjugates may have been able to enter the virion particles and bind to the TAR region of the viral RNA. The PNA-CPP may also have altered or disrupted the viral envelope through the interaction of the CPP moiety with the viral lipid bilayer, thereby inhibiting host cell infection. The authors showed that brief exposure of virions to CPP-PNA conjugates blocked endogenous reverse transcription within virion particles, arguing in favor of a virucidal effect due to the PNA moiety interaction with the viral RNA. However, their work set the stage for exploring anti-HIV-1 activity displayed by CPPs on their own.

CPPs share structural similarities and membrane-binding properties with another class of peptides: the antimicrobial peptides (AMPs) [31]. AMPs are small peptides carrying basic and hydrophobic residues. They are found in all living organisms. Mainly produced by leukocytes and epithelial cells, they are major players in the innate immune response against infectious agents. Interestingly, AMPs have been put forward as one potential class of novel antivirals. Their antimicrobial activity has been reported against both enveloped and non-enveloped viruses, blocking viral infection in different ways [110]: they can directly inactivate the virion through disruption of its envelop or interaction with its glycoproteins; they can act on infected cells possibly through interactions with cell receptors resulting in alterations in cell-signaling pathways required for virus replication; they can block the fusion of the viral membrane with the endosome of the host cell, thereby interfering with viral replication; finally, they can interact with and inhibit viral enzymes essential to the virus replication [111]. In view of the similarities between CPPs and AMPs and of the reports of anti-HSV-1 effects of some peptides, CPP’s potential intrinsic antiviral activity undoubtedly deserves further investigation. 

## 6. Conclusions

The above reviewed studies contain a common message: CPP-conjugated antiviral agents hold great promise as novel preventive and therapeutic modalities against viral infections. The remarkable antiviral effects of PPMOs against lethal infections with EBOV, VEEV, FLUAV and DENV, and against the usually-lethal infections with PV1 and WNV, call for a wider use of CPPs in the delivery of antiviral drugs. CPP-conjugated antivirals also show promise in inhibiting the reactivation from latent infections of viruses such as HSV-1. Moreover, the assessment of CPP antiviral properties and the understanding of their molecular mechanisms could lead to the exploitation of these activities for the development of microbicides to inactivate virions after exposure.

These CPP-antiviral studies illustrate the search for the “perfect” CPP-cargo couple, as CPP carrier efficiency is dependent on the nature of its cargo, affecting the antiviral activity. This is particularly true for PPMO research where much effort has been put towards developing highly active peptides to outperform the more traditionally used penetratin and tat peptides [61,62]. Improving CPP endosomolytical properties to intensify antiviral activity of the conjugated cargo has become a main focus, as the endosomal escape appears to be critical to antiviral activity [30]. 

CPP should not only promote the cellular uptake of the cargo but also improve its intracellular trafficking to ensure that it reaches the appropriate cellular compartment. This aspect cannot be overlooked when designing antivirals as different viruses may carry their replication cycles in different subcellular locations such as the cytosol, the nucleus or in an intricate membrane system within the cytosol. The type of linkage between the CPP and the antiviral compound is of importance as for instance a disulphide bond would result in the rapid release of the cargo through the action of cytoplasm glutathione. Therefore the choice of CPP should not only be optimized to the cargo but should also be specific to the virus to ensure that the antiviral molecule efficiently reaches its viral target in the subcellular compartment.

Research on antiviral CPP-PNA, -siRNA and -protein conjugates, although encouraging, is still at its infancy and importantly needs validation in animal models. There is a need for improvement in the design of CPP to tackle the issue of *in vivo* toxicity and thereby make the drug conjugates safer. As Stein highlights, even though PPMO dose regimens are shown to be nontoxic, it will be necessary to show that treatment with doses several times higher than the expected therapeutic dose is well tolerated [60]. It is noteworthy that all the PPMO studies were done in collaboration with a company specializing in RNA-based drugs, AVI BioPharma Inc, which first developed the PMO and provided the PPMO compounds. Of all the CPP-antiviral investigations, PPMO studies are the ones that are at the most advanced stage, with several reports of use in animal models and directed against a wide range of viruses. The work on PPMO illustrates the successful collaboration between academia and industry. 

One recurring challenge in the global control of viral infections is the development of drug-resistant viral strains. It is very likely that escape mutants will arise during treatment, especially if long-term therapy is required. This issue can be overcome if treatment can be easily switched to different antisense molecules. The ease to synthesize a new CPP-PNA, CPP-siRNA or PPMO directed to the mutated sequence needs to be addressed. According to Stein, PPMO production is costly and complex [60]. Most investigators limited their *in vivo* studies to the most potent PPMO identified in cell culture. However, treatment with a combination of antisense molecules targeting multiple sequences in the viral genome should have a greater antiviral effect compared to a treatment with a single antisense molecule, and should prevent selection of escape variants. More studies with treatment with a cocktail of antivirals would be of critical relevance for further clinical applications.

A number of CPPs are derived from viral proteins, such tat, VP22, and FHV. The role of these cell penetrating motifs during infection has yet to be elucidated but it is interesting to see that another obligate intracellular pathogen, *Mycobacterium tuberculosis*, also possesses a cell wall protein with such a peptide sequence [25]. CPPs represent an example of how understanding how pathogens interact with human host cells can lead to new ways to treat and prevent viral infections.
